# Non-thermal atmospheric pressure plasma treatment increases hydrophilicity and promotes cell growth on titanium alloys in vitro

**DOI:** 10.1038/s41598-023-41905-9

**Published:** 2023-09-08

**Authors:** Woo-Lam Jo, Young-Wook Lim, Soon-Yong Kwon, Ji-Hoon Bahk, Jungsung Kim, Taejin Shin, YongHwa Kim

**Affiliations:** 1 Department of Orthopaedic Surgery, Seoul St. Mary’s Hospital, College of Medicine, The Catholic University of Korea, Seoul, 222, Banpo-daero, Seocho-gu Korea; 2 Central R&D Center, Corentec Co., Ltd., 33-2, Banpo-daero 20-gil, Seocho-gu, Seoul, Korea

**Keywords:** Biomedical engineering, Surface patterning

## Abstract

Most medical implants are made of titanium. When titanium is exposed to air for a long time, hydrocarbons are deposited and the surface becomes hydrophobic. Cell attachment is important for bone ingrowth to occur on the implant surface, and hydrophilicity can enhance this. We examined whether non-thermal atmospheric pressure plasma treatment could increase the hydrophilicity of the titanium surface. Samples coated with four widely used coating types [grit blasting, micro arc oxidation (MAO), titanium plasma spray (TPS), and direct metal fabrication (DMF)] were treated with plasma. Each of the four surface-treated samples was divided into groups with and without plasma treatment. We analysed wettability by surface analysis and evaluation of contact angles, cell proliferation, and adhesion using scanning electron microscopy (SEM), confocal laser scanning microscopy, absorbance tests, and alkaline phosphatase (ALP) activity assay; four different Ti_6_Al_4_V surface types were compared. After plasma treatment, the contact angle was reduced on all surfaces, and the carbon content was reduced on all surfaces based on X-ray photoelectron spectroscopy (XPS) surface analysis. Under confocal laser scanning, the cell layer was thicker on the plasma-treated samples, especially in groups TPS and DMF. Cell proliferation was 41.8%, 17.7%, 54.9%, and 83.8% greater for the plasma- than non-plasma-treated grit blasting, MAO, TPS, and DMF samples, respectively. Hydrophilicity increased significantly under plasma treatment, and biological responsivity was also improved.

## Introduction

Surface coating enhances bone growth on implant surface ^1,2^. There are several ways to ensure bone grows well on titanium surfaces. The most commonly used method is to treat the titanium surface with various coatings ^1,3^.

Improvements in coating technology allow rapid osseointegration to improve the stability of the artificial joint ^3^. This facilitates early ambulation after arthroplasty surgery. Hydrocarbons are formed on the surface of implants exposed to air for a long time, resulting in lower hydrophilicity. This process is called “biologic aging”; with aging, hydrophilicity and cell adhesion to the surface decrease ^4^. Plasma surface treatment can slow biologic aging by increasing hydrophilicity and removing hydrocarbons from the implant surface ^5^.

Plasma is derived via the phase transformation of a gas (by gaining energy), yielding a collection of free radicals, electrons, electrically excited atoms, and neutrons ^6,7^.

By removing hydrocarbons through UV irradiation, hydrophilicity can be increased. UV treatment is a simple and cost-effective process that uses a relatively uncomplicated device, but requires a long time. When plasma is generated at room temperature and applied to the implant surface, an increase in polarity surface energy and hydrophilicity can be obtained quicker than with UV treatment. Various attempts have been made to increase the hydrophilicity of dental implants, with encouraging results ^5–8^.

We hypothesised that orthopaedic devices could be treated with plasma applied to improve the osseointegration capacity of cementless implants. We evaluated the contact angles, surface, cell proliferation, and adhesion in each group using scanning electron microscopy (SEM), confocal laser scanning microscopy, absorbance tests, and alkaline phosphatase (ALP) activity assay.

## Methods

### Sample preparation

We made titanium disks (radius 12.7 mm, thickness 5 mm) and applied four types of surface coating to them: grit blasting, micro arc oxidation (MAO), titanium plasma spray (TPS), and direct metal fabrication (DMF). The samples with the four surface coatings were subdivided into groups with and without plasma treatment. Eighty samples were prepared (10 per experiment [eight experiments]).

Grit blasting of the Ti6Al4V discs was performed using 200–500 μm Al_2_O_3_ particles moving in a high-velocity air stream (KSSA-5FD; Kumkang Tech., Korea). The roughness of grit-blasted discs was in the range of ~ 5–7 μm.

MAO treatment of Ti6Al4V discs was carried out in an aqueous electrolyte containing Ca and P; a pulsed DC field (Auto Electro-Mechanics, Korea) was applied. A solution of 0.15 mol/L calcium acetate monohydrate (DaeJung Chemicals, Korea) and 0.02 mol/L glycerol phosphate calcium salt (DaeJung Chemicals) was used as the electrolyte. The applied voltage, frequency, and duty of the pulsed DC power were 250 V, 660 Hz, and 10%, respectively. All MAO treatments were carried out in a water-cooled glass bath for 3 min using a stainless steel plate as a counter electrode.

For the TPS group, Ti6Al4V discs were treated with TPS coating (Ceramed, Portugal) of approximately 500 µm thickness. Ti powder was injected into a plasma gas stream and heated to 20,000 °C using high kinetic force. The powder was shot onto the substrate and melted to form a porous structure.

For the DMF group, pure Ti powder (CPTi powder grade 2, ASTM F1580, (45–150 µm; GE Additive, USA) was melted and laminated by applying a selective laser to the Ti6Al4V disc surface. Initially, a computer-aided design (CAD) program (Siemens NX CAD/CAM software; Siemens, Germany) was used to design a porous structure identical to cancellous bone ^9^.

### Plasma treatment

**Figure 1:**
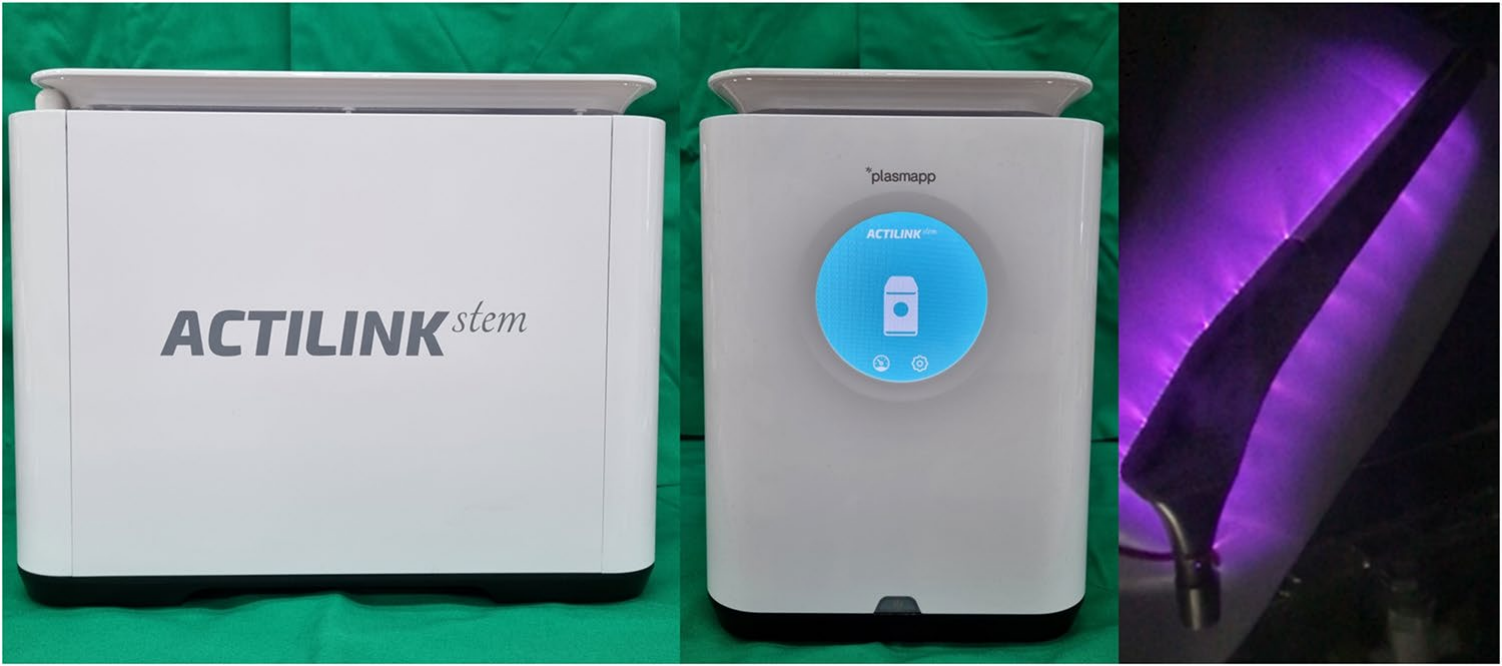
Plasma treatment equipment.

Plasma treatment was applied for 135 s (ACTILINK; Plasmapp, Korea) (Fig. 1). A capacitively coupled plasma (CCP) is discharged in a vacuum chamber. The vacuum chamber is made of dielectric material of Polypropylene and it is also used as a dielectric barrier layer for discharging plasma. A sinusoidal electric power with a frequency of 100 kHz and voltage of 3 kV was applied to the bottom electrode while the top electrode is electrically grounded when the base pressure of 5 torrs is obtained by using a diaphragm vacuum pump. The electron temperature is expected to be about 1–5 eV. For the first 40 s (initial pumping), the pressure was ≤ 10 mmHg; the treatment then continued for a further 85 s. Plasma was processed at room temperature under low pressure. The plasma source was a high-voltage power supply with dielectric barrier discharge (DBD). The voltage was 2.5 ± 0.2 kV and the frequency was 105 ± 5 kHz. The maximum output power was set to 20 W. The process was completed by venting to atmospheric pressure for 10 s.

### Contact angle measurement

The contact angle was measured using a contact angle analyser (Gitsoft, Korea) according to the manufacturer's instructions ^10^. We measured the contact angle within 5 min after treatment in the plasma treatment machine.

### X-ray photoelectron spectroscopy (XPS) analysis

After coating the test specimens, the chemical composition and porosity of the two surfaces were examined using energy-dispersive x-ray spectroscopy (K-alpha plus; Thermo Fisher Scientific, USA). The beam size was 400 μm, and monochromatic Al Kα x-rays produced photons with 1486.6 eV energy. Wide (step size, 1000 meV; pass energy, 200 eV; dwell time, 200 ms; range: 1400 to − 10 eV) and narrow (step size, 100 meV; pass energy, 50 eV; dwell time, 500 ms) regions were scanned for each sample.

### Human blast cell culture

We purchased primary human osteoblasts (C-12720; PromoCell, Germany) isolated from femoral trabecular bone tissue of the knee or hip joint. The cells were cultured in growth medium (C-27001; PromoCell, Germany) and kept at 37 °C for 24 h in a humidified incubator under 5% CO_2_. The medium in the culture plates was replaced every 3 days, with a total incubation period of 15 days.

### Cell morphology (scanning electron microscopy)

Osteoblasts were seeded at a density of 1.5 × 10^4^ cells for all specimens. After 4 h, the media was removed and the cells were washed thrice with phosphate-buffered saline (PBS). After adding 2% glutaraldehyde-PBS solution, the cells were stabilised for 2 h, and then washed with distilled water. The cells were dehydrated using 50–100% ethanol at 30-min intervals. Ethanol was then removed, and the cells were left to stand at room temperature to allow for complete ethanol evaporation. The surfaces were observed using the Verios G4 UC extreme high-resolution scanning electron microscope (Thermo Fisher Scientific).

### Confocal laser scanning microscopy

We cultured human osteoblasts on each sample surface, seeded at a density of 1.5 × 10^4^ cells. The cells were stained using PHK-26 (MINI26; Sigma-Aldrich, USA) and cultured on the sample surfaces. To stain the nuclei blue, a mounting medium with DAPI (4', 6-diamidino-2-phenylindole) (VECTASHIELD, H-1200; Vector Labs, USA) was used.

After 4 h of incubation, cell morphology was observed using a confocal laser scanning microscope (LSM900; Carl Zeiss, Germany).

After 6 days of culture, the cells were washed thrice with PBS and fixed with 3.7% (v/v) paraformaldehyde at room temperature for 15–20 min. The preserved cells were irrigated with PBS thrice, treated with 0.1% Triton X-100 for 10 min, and irrigated again with PBS. Rhodamine-phalloidin (Molecular Probes, USA) was diluted to 1:100 and left to react for 1 h at 37 °C without any light exposure. It was then sprayed with PBS three times, mounted in aqueous mount and examined using a confocal microscope.

### Human osteoblast cell culture

Primary human osteoblasts were seeded at a density of 1.5 × 10^4^ cells for all specimens and incubated for 4 h, 24 h, 5, 10, or 15 days. All samples were reacted with WST-8 reagent (ab228554; Abcam, UK). Cell proliferation was assessed through absorbance measurements (PowerWave XS microplate spectrophotometer; Bio-Tek Instruments, USA).

### Statistical analysis

Mean cell proliferation and ALP activity were analysed using a paired t-test for parametric data and the Mann–Whitney U test for non-parametric data. Statistical analysis was performed using SPSS Statistics 21.0 software (IBM, USA).

### Ethics approval

Since this is basic research, it did not require ethics approval.

### Approval for human experiments

We purchased primary human osteoblasts from PromoCell.

## Results

**Figure 2:**
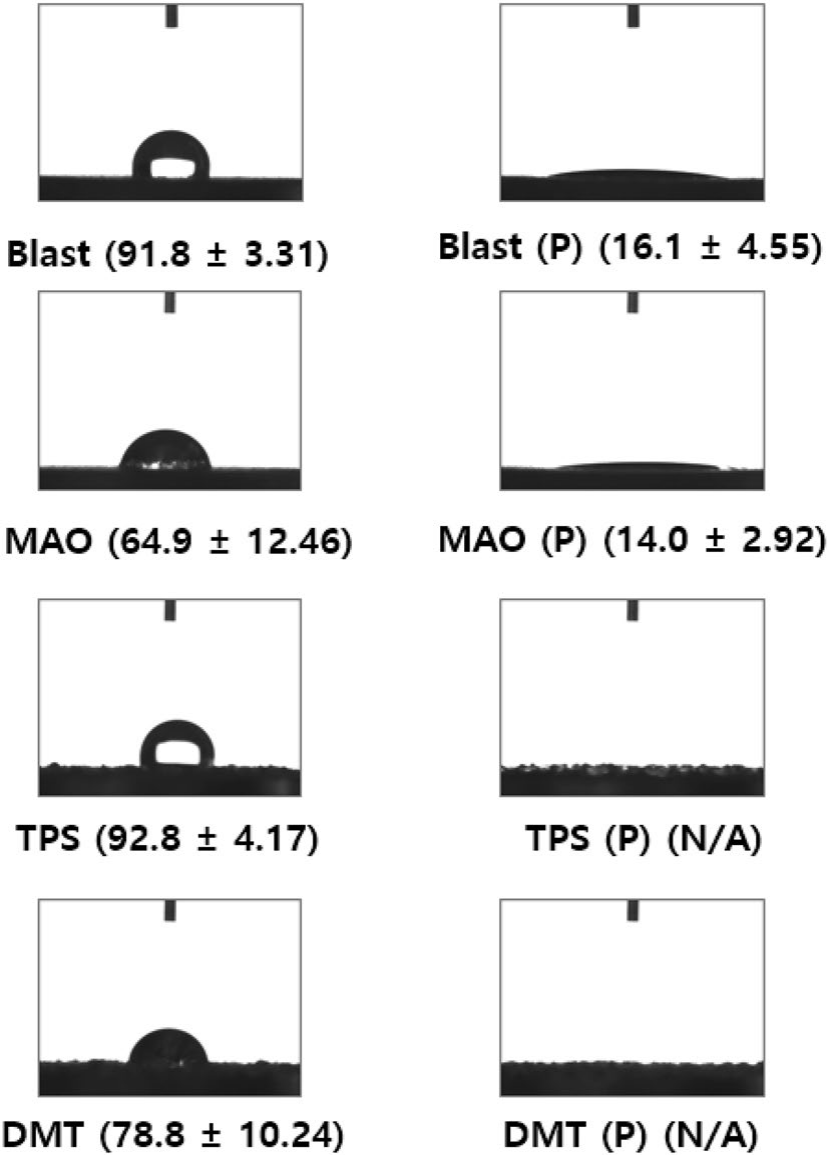
The contact angle decreased after plasma treatment, as shown in the right-hand images. The numbers in parentheses are the mean ± SD contact angle (°). *SD* standard deviation, *MAO* micro arc oxidation, *TPS* titanium plasma spray, *DMF* direct metal fabrication, *N/A* not available.

The contact angle was significantly reduced in the plasma-treated group. In the grit blasting group, the average contact angle was 91.8° for non-plasma treated samples, but decreased to 16.1° for plasma-treated samples. In the MAO-coated group, the average contract angle was 64.9° for the non-plasma treated samples, but decreased significantly to 14° for the plasma-treated samples. In the TPS- and DMF-coated groups, plasma treatment completely spread the fluid over the surface, so the contact angle could not be measured (Fig. 2).

**Figure 3:**
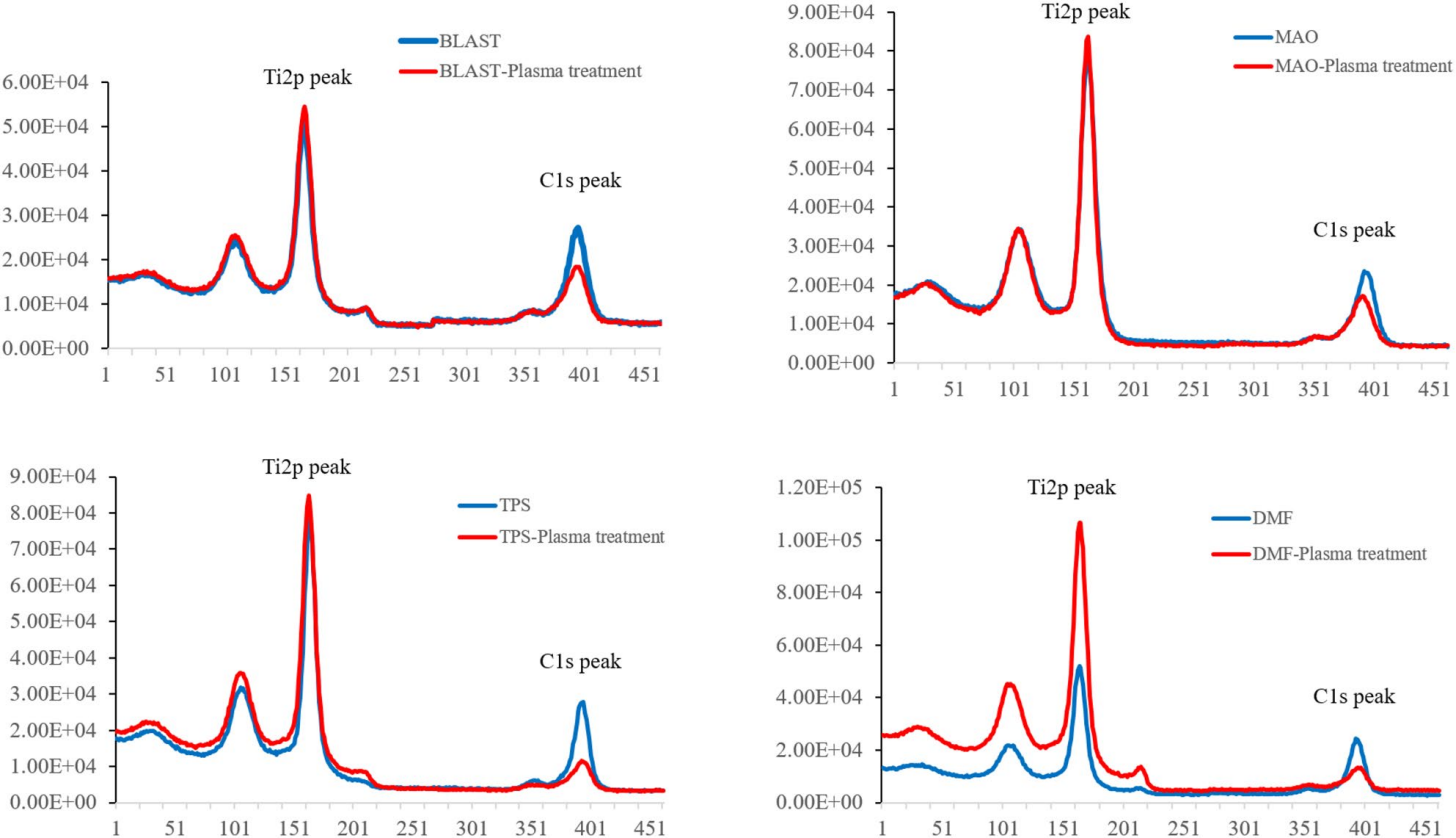
High-resolution Ti2p and C1s peaks obtained by XPS. The Mann–Whitney U test used to make comparisons.

According to XPS analysis, the carbon content was low in all groups subjected to plasma treatment. In particular, the C1s peaks in the TPS- and DMF-coated groups were reduced by 40–56% after plasma treatment (Fig. 3).

**Figure 4:**
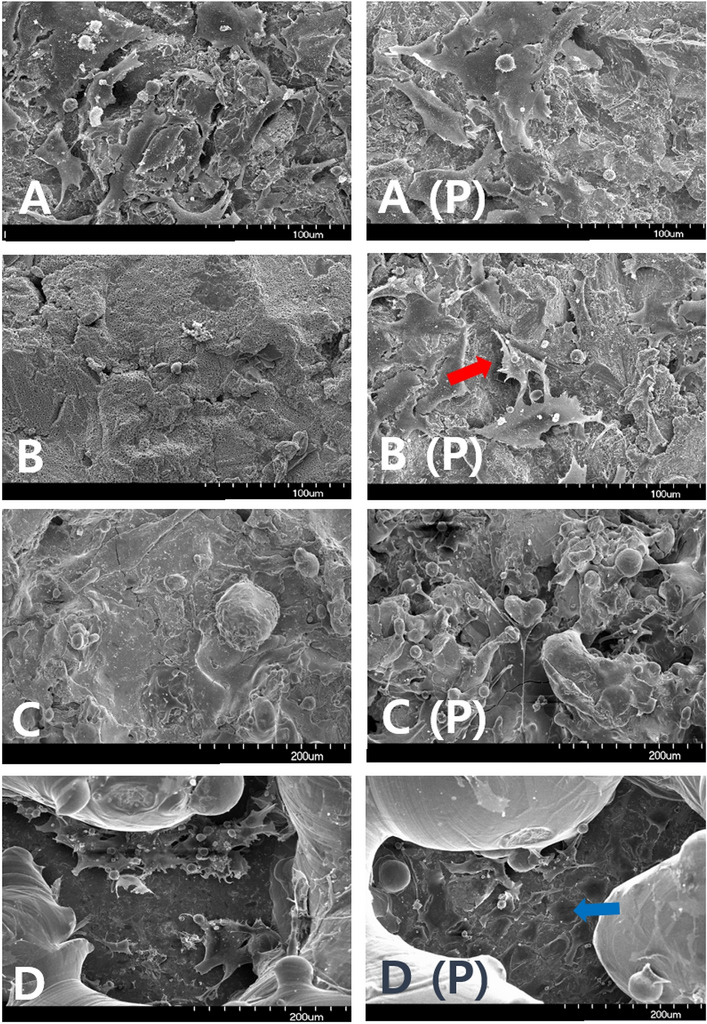
Scanning electron microscopy images of samples subjected to (**A**) grit blasting (× 500), (**B**) MAO (× 500), (**C**) TPS (× 250), and (**D**) DMF (× 250) without (left) and with plasma treatment (right). MAO samples with plasma-treated surfaces were covered with healthy osteoblasts and lamellipodia (red arrow), while thin cytoplasmic projections were observed in plasma-treated DMF samples (blue arrow).

We inspected the coatings with and without plasma treatment using SEM. There were no differences in cell number, distribution, or shape between the plasma- and non-plasma-treated samples subjected to grit blasting. However, MAO samples with plasma-treated surfaces were covered with healthy osteoblasts and lamellipodia. Moreover, thin cytoplasmic processes branched out from the filopodia and entered the pores after 4 h of proliferation. The morphologies of the two TPS-coated groups were similar. In DMF samples not subjected to plasma treatment, a few cells were scattered on the titanium base, while in the group treated with plasma, the cells were widely spread and filled the base. Thin cytoplasmic projections extended into the DMF surface pores after 4 h of proliferation (Fig. 4).

**Figure 5:**
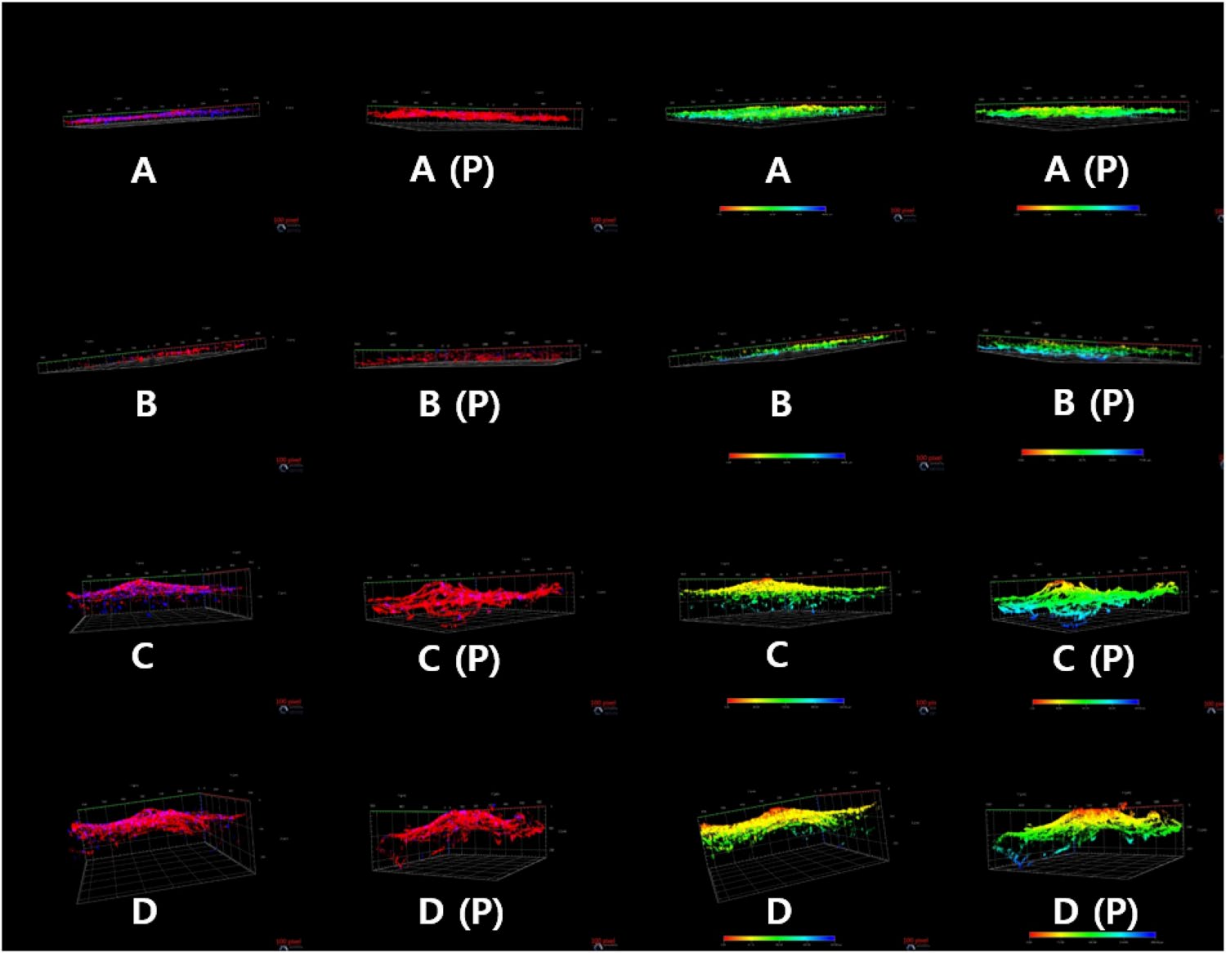
Confocal laser scanning microscopy images, obtained at 6 days, of (**A**) samples subjected to grit blasting, (**B**) MAO, (**C**) TPS, and (**D**) DMF without (left) and with plasma treatment (right). In the first and second columns, the proliferation of actin fibres is superior in the plasma-treated groups. In the third and fourth columns, the distribution and spread of cells are indicated by colour. Cells were widely spread in the plasma-treated samples. Blue indicates deeper cell penetration.

Under confocal laser scanning microscopy, there were no differences between plasma- and non-plasma-treated samples at 4 h, but differences appeared after 6 days. Actin fibres were more active in all plasma-treated groups. We converted images of actin and nucleus staining into colour images based on depth using a computer program. The cell layer was thicker in plasma-treated samples, especially in the TPS and DMF groups (Fig. 5). The osteoblasts were widely distributed in the vertical plane, suggesting that the cells penetrated the coating surfaces, which may be negatively charged.

We analysed cell proliferation over 15 days. Gradually, the difference between groups treated with and without plasma became more obvious. There were no significant differences up to day 5 of proliferation, but on day 10, significant differences were observed among the grit blasting, TPS, and DMF groups.

**Figure 6:**
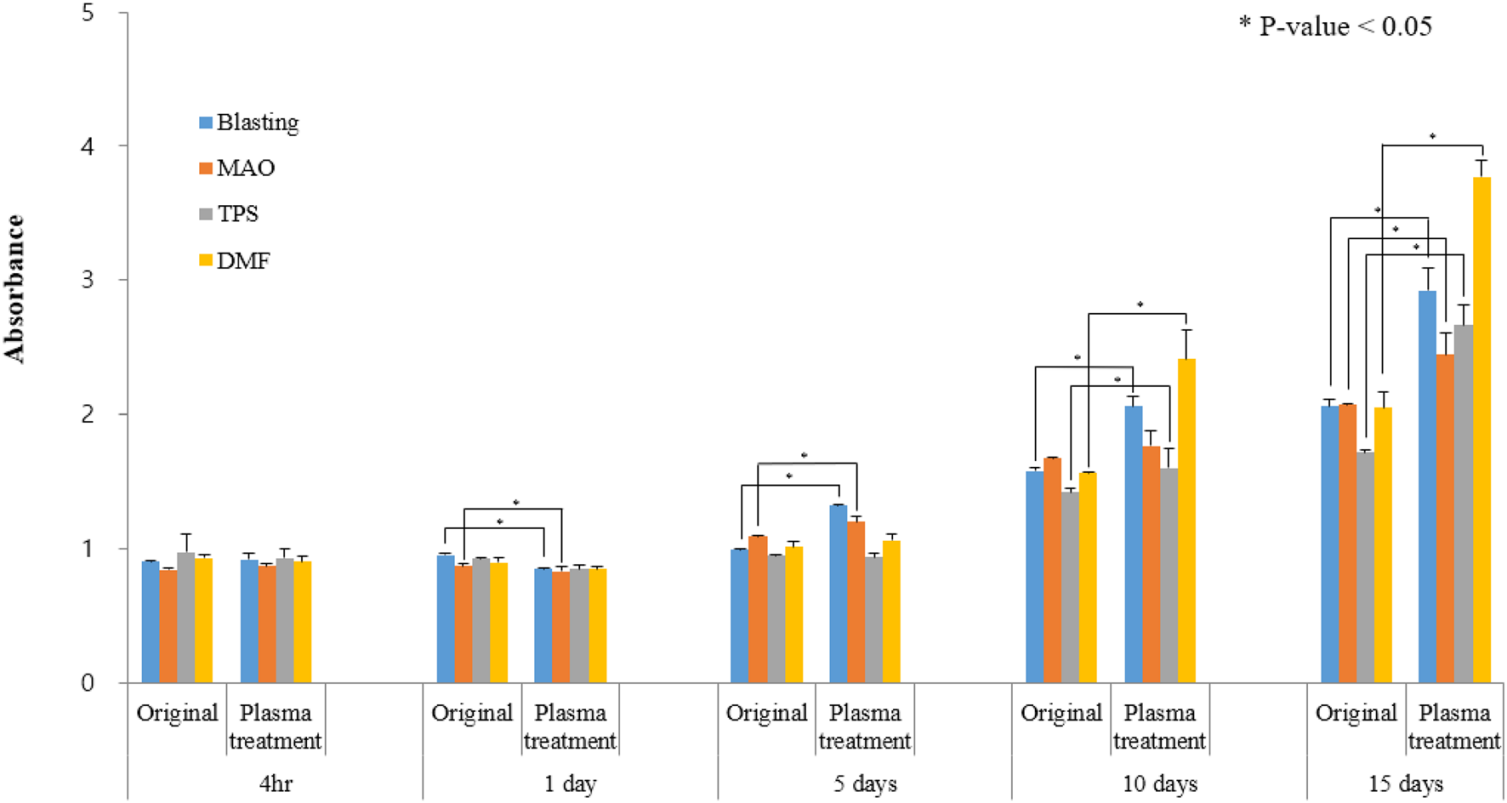
Cell proliferation increased over time in all groups treated with plasma (Mann–Whitney *U* test).

On day 15, the absorbance values were 2.06 ± 0.21 and 2.93 ± 0.07 for the non-plasma- and plasma-treated grit blasting sample surfaces, 2.07 ± 0.01 and 2.45 ± 0.06 for the non-plasma- and plasma-treated MAO surfaces, 1.72 ± 0.01 and 2.66 ± 0.06 for the non-plasma- and plasma-treated TPS surfaces, and 2.05 ± 0.05 and 3.77 ± 0.05 for the non-plasma- and plasma-treated DMF surfaces, respectively; all of the differences between plasma- and non-plasma-treated samples were statistically significant. Cell proliferation was 41.8%, 17.7%, 54.9%, and 83.8% greater for the plasma- than non-plasma-treated grit blasting, MAO, TPS, and DMF samples, respectively (Fig. 6). The highest absorbance was observed for the plasma-treated DMF surfaces, indicating that these cells show the most proliferation.

**Figure 7:**
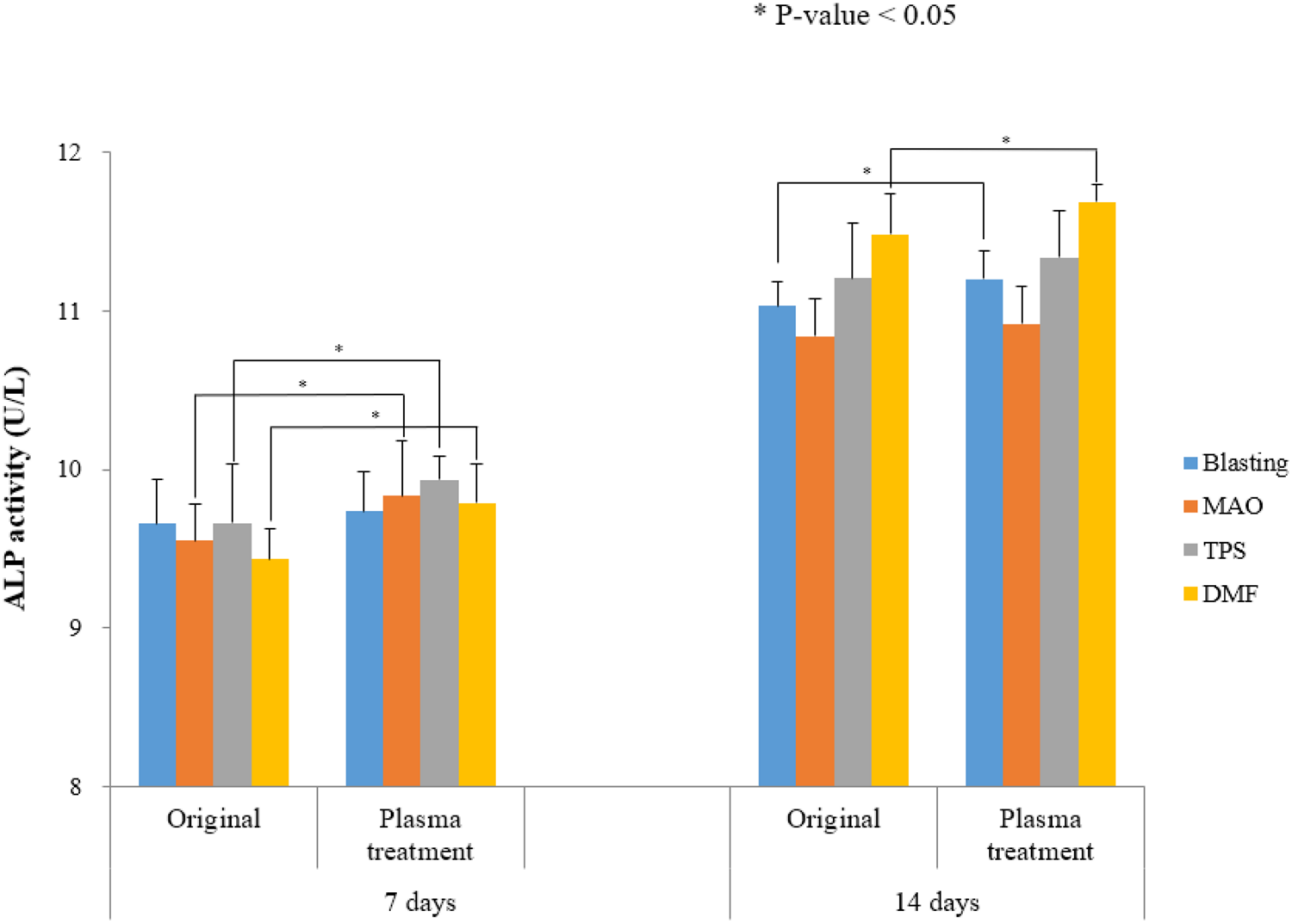
ALP activity was higher in the plasma- than non-plasma-treated grit blasting and DMF samples at 14 days (Mann–Whitney *U* test).

ALP activity was 10.84 ± 0.09 and 10.93 ± 0.10 U/L for the non-plasma- and plasma-treated grit blasting sample surfaces, and 11.46 ± 0.11 U/L and 11.69 ± 0.48 U/L for the non-plasma- and plasma-treated DMF surfaces, respectively. The differences in the other groups were not statistically significant. ALP expression was increased 1.53% and 1.17% in the plasma- treated grit blasting and DMF samples, respectively (Fig. 7).

## Discussion

In this study, we examined the effect of plasma treatment on various coated implant surfaces. The initial attachment and proliferation of human osteoblast cells was enhanced by plasma treatment, regardless of the type of surface coating.

In general, organic impurities, such as hydrocarbons, are always attached to titanium surfaces, which increases hydrophobicity and leads to the degradation of titanium ^11,12^. Titanium continues to degrade between 4 weeks and 6 months of aging ^4^.

As the hydrocarbon level increases, the rates of protein adsorption and cell adhesion decrease. Cell adhesion and ALP activity are increased on hydrophilic compared to hydrophobic surfaces ^13,14^. Moreover, more cells grow on hydrophilic surfaces ^15–17^.

In this study, we confirmed that the hydrophilicity in the plasma treatment groups increased according to the decrease in contact angle. The water contact angle of non-plasma treated surfaces was 64.9°–92.8°. After plasma treatment, it decreased significantly, to almost 0°, on TPS and DMF surfaces. This indicates that the plasma treatment dramatically increased the hydrophilicity of all metal surfaces tested. This hydrophilicity is lost by the degradation of titanium over time. In a previous study, the new titanium surface was superhydrophilic and it became increasingly hydrophobic after 1, 3, and 6 months.

XPS revealed reduced surface carbon content in plasma-treated samples, indicating that the plasma treatment evaporated hydrocarbon organic materials as H_2_O and CO_2_. We confirmed that the carbon content was lower on the metal surface. To our knowledge, this study is the first to measure the carbon content after plasma surface treatment. Specifically, it was very low in the TPS- and DMF- coated groups, which is presumed to be due to the greater surface area of these coatings compared to the other coatings.

SEM inspection confirmed that the cells penetrated deep into the DMF coating and grew. This occurred because the DMF coating is thicker than the other coatings and because cells can migrate to deeper places quickly when hydrophilicity is achieved. These results were confirmed by confocal laser scanning.

Plasma is a collection of free radicals, electrons, electrically excited atoms, neutrons, and protons. For industrial use, specific gases and solids are placed into a chamber under vacuum, and a high voltage is applied to form plasma. This plasma has a very high temperature, and the use of this method for plasma generation is limited due to the large size of the generator. Recently, however, room temperature atmospheric-pressure plasma was developed. When atmospheric pressure plasma is irradiated on the surface of a biomaterial, the surface properties are modified due to a “bombing” effect of reactive species. Here, the principle is to remove hydrocarbons, which are present as a fine dust on the outermost surface, to expose the outermost layer of titanium, i.e. TiO_2_^5^. By removing hydrocarbons on the outer surface of titanium implants, the biological properties of the TiO_2_ layer can be exploited for dentistry applications ^5,7^.

One study reported the application of room temperature atmospheric pressure plasma, using nitrogen (N_2_) for surface treatment of SLA-treated titanium dental implants. Osteoblast adhesion, proliferation, and differentiation on the titanium surface were increased compared to the untreated group ^18^.

Grit blasting, MAO coating, and TPS coating are widely used for orthopaedic implants ^19–21^. DMF involves the application of an additive through laser welding using sprayed titanium powder. It has extremely high mechanical strength (similar to that of forged metal), which makes it suitable for orthopaedic implants ^22,23^. DMF surfaces have a remarkably porous structure. Previous studies reported an average pore size of 200–500 μm, average porosity of 65 ± 5%, and average thickness of 500 ± 100 μm ^22^.

Previously, Suh et al.^24^ found that the sample surfaces had biological and mechanical properties similar to those of the plasma-sprayed surfaces herein, both in vivo and in vitro. DMF coating has greater porosity and thickness, which promotes cell proliferation. In the plasma-treated DMF coated samples, confocal laser scanning microscopy demonstrated that the cell distribution was most diffuse through 286 µm; these samples also showed the highest absorbance and cell proliferation rates.

Confocal laser scanning microscopy demonstrated a wider distribution of cells in plasma- than non-plasma-treated TPS and DMF samples. Plasma treatment increased TPS and DMF sample cell proliferation by 54.9% and 83.8%, respectively. This suggests that plasma treatment is more effective for ingrowth (TPS and DMF) than ongrowth (MAO and grit blasting) coating methods.

A limitation of this study was the small sample size. Nevertheless, statistically significant results were obtained using nonparametric tests. Furthermore, not all cells could not be harvested from metal because of the surface roughness, although we attempted to overcome this by increasing the amount of the media. And we do not have optical emission spectrum (OES) of plasma. Further investigation will be performed to understand the underlying physics and mechanism of plasma reaction on the implant surface by using probe and OES. As a final limitation, no DNA analysis, such as Western blotting, was performed. As this was an in vitro study, we are planning further in vivo investigations. We plan to conduct an in vivo study to confirm rapid osseointegration with plasma treatment and to conduct cytotoxicity tests of degradable substances that may be produced during the plasma treatment.

In conclusion, plasma treatment was applied to samples coated via four coating types that are currently used widely. Hydrophilicity increased significantly under plasma treatment, and biological responsivity was thus also improved. The application of this technique in artificial joint surgery may reduce the initial time required to achieve implant stability.

## Data Availability

The datasets generated and analysed in the current study are available from the corresponding author on reasonable request.
